# Better operative outcomes achieved with the prone jackknife vs. lithotomy position during abdominoperineal resection in patients with low rectal cancer

**DOI:** 10.1186/s12957-015-0453-5

**Published:** 2015-02-12

**Authors:** Peng Liu, Haidong Bao, Xianbin Zhang, Jian Zhang, Li Ma, Yulin Wang, Chunyan Li, Zhongyu Wang, Peng Gong

**Affiliations:** Department of General Surgery, The First Affiliated Hospital of Dalian Medical University, 222 Zhongshan Road, 116011 Dalian, China; Department of Epidemiology, Dalian Medical University, 9 Lvshun Road South, 116044 Dalian, China; Department of Gastroenterology, The First Affiliated Hospital of Dalian Medical University, 222 Zhongshan Road, 116011 Dalian, China

**Keywords:** Rectal cancer, Abdominoperineal resection, Lithotomy position, Prone jackknife position

## Abstract

**Background:**

Lithotomy (LT) and prone jackknife positions (PJ) are routinely used for abdominoperineal resection (APR). The present study compared the clinical, pathological, and oncological outcomes of PJ-APR vs. LT-APR in low rectal cancer patients in order to confirm which position will provide more benefits to patients undergoing APR.

**Methods:**

This is a retrospective study of consecutive patients with low rectal cancer who underwent curative APR between January 2002 and December 2011. Patients were matched 1:2 (PJ-APR = 74 and LT-APR = 37 patients) based on gender and age. Perioperative data, postoperative outcomes, and survival were compared between the two approaches.

**Results:**

Hospital stay was shorter with PJ-APR compared with LT-APR (*P* < 0.05). Compared with LT-APR, duration of anesthesia (234 ± 50.8 vs. 291 ± 69 min, *P* = 0.022) and surgery (183 ± 44.8 vs. 234 ± 60 min, *P* = 0.016) was shorter with PJ-APR, and estimated blood losses were smaller (549 ± 218 vs. 674 ± 350 mL, *P* < 0.001). Blood transfusions were required in 37.8% of LT-APR patients and in 8.1% of PJ-APR patients (*P* < 0.001). There was no difference in the distribution of N stages (*P* = 0.27). Median follow-up was 47.1 (13.6–129.7) months. Postoperative complications were reported by fewer patients after PJ-APR compared with LT-APR (14.9% vs. 32.4%, *P* = 0.030). There were no significant differences in overall survival, disease-free survival, local recurrence, and distant metastasis (*P* > 0.05).

**Conclusions:**

The PJ position provided a better exposure for low rectal cancer and had a lower operative risk and complication rates than LT-APR. However, there was no difference in rectal cancer prognosis between the two approaches. PJ-APR might be a better choice for patients with low rectal cancer.

## Background

Abdominoperineal resection (APR) remained for many years the main treatment option for most patients with low rectal cancer, despite high local recurrence and poor prognosis [1-5]. APR was gradually replaced by anterior resection (AR) and low anterior resection (LAR) for tumors of the upper and middle rectum [6]. However, APR is still required in selected cases of low rectal cancer [7-9].

Lithotomy (LT) and prone jackknife (PJ) positions are the two main positions currently used in APR. The LT position provides adequate access to the rectovaginal septum and allows easy access to the posterior face of the rectum [10]. However, this position is uncomfortable for the surgeons and the assistants, blood tends to accumulate in the operation area, and adequate lighting is often challenging. The PJ position is appropriate for almost all proctological surgeries. It allows an excellent exposure of the posterior and anal perineum and the anterior face of the rectum, provides a more comfortable position for the surgeon and assistants, results in less blood accumulation, and enables better lighting. Specimens are also more easily obtained, with less circumferential resection margin (CRM) involvement than with the LT position [11,12]. A previous study has suggested that the prone position results in shorter operative time and in lower incidence of perineal wound infection, but the researchers did not look at the oncological outcomes [13]. Another study has suggested that there might be no difference in perioperative morbidity and oncological outcomes between the PJ and LT positions [14].

Therefore, the oncological outcomes are still in need of a better assessment. The objective of the present study was to compare these two approaches in terms of operative and oncological outcomes, in order to assess the impact of the position on the operative and oncological outcomes of patients undergoing APR.

## Methods

### Study population

In this retrospective study, we included all consecutive patients who underwent radical APR for stages I to III low rectal cancers at the First Affiliated Hospital of Dalian Medical University between January 2002 and December 2011. Inclusion criteria were 1) low rectal cancer, 2) absence of distant metastases, and 3) APR aiming to curative resection (R0).

Exclusion criteria were 1) inflammatory bowel diseases, 2) hereditary colorectal cancer syndromes, 3) recurrent cancer after anus-preserving surgery, 4) non-rectal cancer history, or 5) lost to follow-up or incomplete follow-up data. No patient received neoadjuvant therapy.

Pre-surgical assessment and staging were made using physical and laboratory examinations, including nutritional status, digital rectal examination, proctoscopy, tumor distance from the dentate line, colonoscopy, serum carcino-embryonic antigen (CEA) levels, chest radiography, chest computed tomography (CT) scan, abdominal and pelvic CT scan, endorectal ultrasound, and/or pelvic magnetic resonance imaging (MRI).

The present study was approved by the ethics committee of the First Affiliated Hospital of Dalian Medical University (# LCKY2012-34), and the need for individual consent was waived.

Because the number of patients was higher in the PJ-APR group (*n* = 192) than in the LT-APR group (*n* = 37), 1:2 matching of eligible patients was performed based on gender and age. Matching was done to decrease the possible bias due to the difference in the number of patients between the groups.

### Data collection

Data were collected from the electronic medical records, including demographic data, clinical and pathological parameters, tumor-node-metastasis (TNM) status, adjuvant chemotherapy and/or radiation therapy, operative parameters, perioperative morbidity and mortality, postoperative outcomes, and oncological outcomes (overall survival, cancer-specific mortality, distant metastases, and local recurrence). TNM staging was based on the seventh edition of the AJCC TNM staging manual.

### Surgical techniques

The operative position was selected by the surgeon according to his preoperative evaluation. All operations were performed by skilled colorectal surgeons with emphasis on the oncological principles of TME.

In PJ-APR, anal closure with purse string sutures was followed by an elliptical cutaneous incision 2 to 3 cm from the anal margin. The subcutaneous fat was divided and ligated laterally to expose both sides of the medial margin of the gluteus maximus muscle. Adipose tissue in the ischiorectal fossa was removed following anal vein ligation. The pelvic floor was identified and opened just anterior to the coccyx. The levator ani were then divided laterally as near as possible to its bony insertion to the pelvic sidewalls beginning from the posterior midline, and the dissection line joined the previously developed presacral plane of dissection.

The perineal wound was closed with a pelvic drain, opening near the incision. Patients were then turned into the supine position. All patients underwent high ligation of the inferior mesenteric artery and complete excision of the mesorectum. Sharp dissection of the mesorectum was performed in the avascular plane between the visceral fascia and the presacral fascia [15]. Care was taken to preserve the preaortic sympathetic plexus and hypogastric nerves. Laterally, the pelvic plexus was also preserved unless there was evidence of tumor invasion; the sacral nerve roots were preserved [8]. A permanent sigmoid colon colostomy was performed.

The first step of LT-APR was performed in the supine position. After establishment of a permanent sigmoid colon colostomy, patients were turned into the LT position, and the perineal dissection was then performed. The dissection was performed along similar planes and with the same goals as in PJ-APR, i.e., dissecting the levator ani entirely from the pelvic sidewalls [14], working circumferentially from posterior to anterior, and resulting in en bloc resection of the tumor and the adjacent pelvic floor without opening the interface between the tumor and the levator ani. Posterior vaginal resection was performed en bloc with the specimen if a tumor involving the anterior rectal wall was adherent to the vagina. Occasionally, partial prostatectomy was required [8].

### Surgical aims

Curative intent was defined as the complete removal of gross tumor and tumor-associated lymph nodes, confirmed by pathological examination. Photographic documentation and the pathological examination of the specimen were performed by at least two pathologists with a special interest in colorectal surgery.

Blood loss was estimated based on the amount of blood in the aspiration devices minus the amount of saline used, and on the amount of blood in the gauzes, estimated using a gravimetric method [16].

### Postoperative follow-up

Patients were followed up at 3-month intervals during the first 2 years, then every 6 months until 5 years. The follow-up was performed by the colorectal surgeons. Routine postoperative examination included a physical examination, blood CEA levels, colonoscopic examinations, chest X-rays, abdominopelvic CT or MRI scan, and/or whole-body bone scan.

Wound infection was defined as an infection of the surgical incision. The infection had to occur within 30 days of surgery, and the culture had to be positive.

Local recurrence within the pelvis was proven by 1) positive histology, 2) diagnostic imaging evidence with raised CEA levels, or 3) macroscopic evidence of tumor recurrence at laparotomy [17]. The presence of a tumor at any other site was defined as distant metastasis.

Disease-free survival was the time from the date of the primary treatment to the date of the first instance of recurrent disease (either local, systemic, or both if they occurred less than 6 months apart). Overall survival was defined from the date of the primary treatment to the date of death from any cause [18].

For the purpose of the study, follow-up was closed on March 31, 2013.

### Statistical analysis

Statistical analysis was performed using SPSS 16 (SPSS Inc., Chicago, IL, USA). Categorical variables were compared using chi-square or Fisher’s exact tests, as appropriate. Continuous variables are presented as means ± SD and were compared using independent samples *t*-tests. Kaplan-Meier curves and log-rank tests were used to evaluate oncological outcomes. Multivariate Cox regression analysis was performed for survival outcome. The dependent variable was survival, and the independent variables included gender, age, surgical position, tumor size, pathological type, tumor differentiation, pathologic T and N stages, vascular tumor embolus, postoperative radiotherapy, and chemotherapy. *P <* 0.05 was considered statistically significant.

## Results

### Patients’ characteristics

Between January 2002 and December 2011, 383 patients underwent curative APR for low rectal cancer; 154 patients were excluded from the analysis, and 229 patients with primary rectal cancer were included. Among them, 192 (83.8%) underwent PJ-APR and 37 (16.2%) underwent LT-APR; 147 were men, and 82 were women. Median age was 65 years (range: 28 to 86).

After matching, there were 37 patients in the LT-APR group and 74 patients in the PJ-APR group. Besides the matching criteria, there were no differences in pTNM stage, postoperative chemo- and radiotherapy, histological grade, pathological type, body mass index, tumor stage, and duration of liquid and semi-liquid diets between the two groups (all *P* > 0.05) (Table 1).

**Table 1:** **Perioperative variables according to patient positioning**

**Variables**		**PJ-APR (** ***n*** **= 74)**	**LT-APR (** ***n*** **= 37)**	***P*** **value**
Gender, *n* (%)	Male	52 (70)	26 (70)	0.591
	Female	22 (30)	11 (30)	
Age, years		63.0 ± 10.5 (39–83)	64.0 ± 11.7 (39–84)	0.396
Body mass index, kg/m^2^, mean (range)		23.2 ± 3.3 (16.4–29.4)	22.9 ± 3.2 (16.8–31.2)	0.581
Length of hospital stay, days		23 ± 7.4 (11–46)	29 ± 11.7 (17–63)	0.011
Duration of anesthesia, min		234 ± 51 (130–385)	291 ± 69 (150–450)	0.022
Operative time, min		183 ± 45 (90–320)	234 ± 60 (120–370)	0.016
Operative time, *n* (%)	≤120	6 (8.1)	1 (2.7)	0.255
	>120	68 (91.9)	36 (97.3)	
Estimated blood loss, mL		549 ± 218 (200–1,137)	674 ± 350 (160–1,440)	<0.001
Blood transfusion, *n* (%)		6 (8.1)	14 (37.8)	<0.001
Tumor distance, cm		4.0 ± 1.4 (1–10)	3.9 ± 1.1 (2–7)	0.282
Tumor size, cm		4.4 ± 1.8 (1.5-12)	4.7 ± 1.3 (2.4–8)	0.221
Macroscopic type of tumor, *n* (%)	Mushroom	19 (25.7)	6 (16.2)	0.486
	Ulcerative	54 (72.9)	30 (81.1)	
	Infiltrative	1 (1.4)	1 (2.7)	
Pathological type, *n* (%)	Adenocarcinoma	73 (98.6)	36 (97.3)	0.286
	Squamous carcinoma	1 (1.4)	0	
	Rectal carcinoid	0	1 (2.7)	
Degree of differentiation, *n* (%)	Good	4 (5.4)	1 (2.7)	0.537
	Moderate	49 (66.2)	28 (75.7)	
	Poor	21 (28.4)	8 (21.6)	
Pathologic T stage, *n* (%)	T1	0	1 (2.6)	0.468
	T2	16 (21.6)	10 (27)	
	T3	54 (73.0)	24 (65)	
	T4	4 (5.4)	2 (5.4)	
Pathologic N stage, *n* (%)	N0	36 (48.6)	20 (54.1)	0.269
	N1	26 (35.1)	15 (40.5)	
	N2	12 (16.2)	2 (5.4)	
Time of fluid diet, days		4.2 ± 1.2 (2–8)	4.0 ± 0.9 (2–7)	0.055
Time of semi-liquid diet, days		7.1 ± 1.9 (4–14)	6.7 ± 3.3 (3–22)	0.386
Pelvic drain time, days		10.5 ± 3.1 (5–22)	9.4 ± 3.8 (5–20)	0.338
Postoperative hospitalization, days		15.6 ± 5.3 (7–35)	22 ± 10.8 (10–51)	<0.001
Postoperative chemoradiotherapy, *n* (%)	Yes	35 (47.3)	15 (40.5)	0.319
	No	39 (52.7)	22 (59.5)	

*APR* abdominoperineal resection, *PJ-APR* abdominoperineal resection in prone jackknife position, *LT-APR* abdominoperineal resection in lithotomy position.

### Perioperative and short-term postoperative outcomes

Anesthesia duration and operative time were both significantly shorter in patients who underwent PJ-APR (anesthesia: 234 ± 51 vs. 291 ± 69 min, *P* = 0.002; operation: 183 ± 45 vs. 234 ± 60 min, *P* = 0.016) (Table 1). In addition, estimated blood loss during PJ-APR was significantly less than during LT-APR (549 ± 218 vs. 674 ± 350 mL, *P* < 0.001), and more patients who underwent LT-APR required blood transfusion (*P* < 0.001) (Table 1). There were no significant differences in the distance of the tumor to the dentate line, tumor size, pathological type, differentiation, and T or N stage (Table 1). The specimens were assessed by at least two pathologists. Results of the pathological examination showed that the surgical margins of the specimens were negative.

Two patients in the PJ-APR group and two in the LT-APR group experienced complications during the operation (*P* = 0.407) (Table 2). No female patient suffered from vaginal injury and hemorrhage during operation. After surgery, 11 (14.9%) of the 74 patients who underwent PJ-APR experienced postoperative complications, compared with 12 (32.4%) of the 37 patients who underwent LT-APR (*P* = 0.030; Table 2). Occurrence of perineal wound infection (1.4%) and incision fat necrosis (1.4%) was significantly lower in the PJ-APR group than in the LT-APR (10.8% and 16.2%, respectively, both *P* < 0.05). There was no difference in the rate of abdominal infection (4.1% vs. 8.1%, *P* = 0.317) (Table 2).

**Table 2:** **Complications of APR according to patient positioning**

**Variables**	**PJ-APR, (** ***n*** **= 74)**	**LT-APR, (** ***n*** **= 37)**	***P*** **value**
Intraoperative complications, *n* (%)			
Total	2 (2.7)	2 (5.4)	0.407
Iatrogenic tumor perforation	2 (2.7)	2 (5.4)	0.407
Vaginal injury	0	0	NA
Urethral/ureteric injury	1 (1.4)	1 (2.7)	0.558
Hemorrhage	0	0	NA
Postoperative complications, *n* (%)			
Total	11 (14.9)	12 (32.4)	0.030
Urinary retention	7 (9.5)	7 (18.9)	0.134
Abdominal wound infection	3 (4.1)	3 (8.1)	0.317
Perineal wound infection	1 (1.4)	4 (10.8)	0.042
Incision fat necrosis	1 (1.4)	6 (16.2)	0.005
Incision dehiscence	1 (1.4)	3 (8.1)	0.107
Intestinal obstruction	0	1 (2.7)	0.333
DVT (deep venous thrombosis)	1 (1.4)	1 (2.7)	0.558
Pelvic abscess	1 (1.4)	1 (2.7)	0.558
Reoperation	1 (1.4)	0	0.667
Readmission	3 (4.1)	2 (5.4)	0.542

*APR* abdominoperineal resection, *PJ-APR* abdominoperineal resection in prone jackknife position, *LT-APR* abdominoperineal resection in lithotomy position, *NA* not applicable, *DVT* deep venous thrombosis.

### Long-term oncological outcomes

By March 2013, 64 (57.7%) patients were alive at a median follow-up of 47.1 (13.6 to 129.7) months. A total of 47 patients (42.3%) died during the study period. The causes of death included local recurrence (*n* = 14), distant metastases (*n* = 22), or both (*n* = 8). Two patients died from heart disease, and the last one died in a car accident.

**Figure 1:**
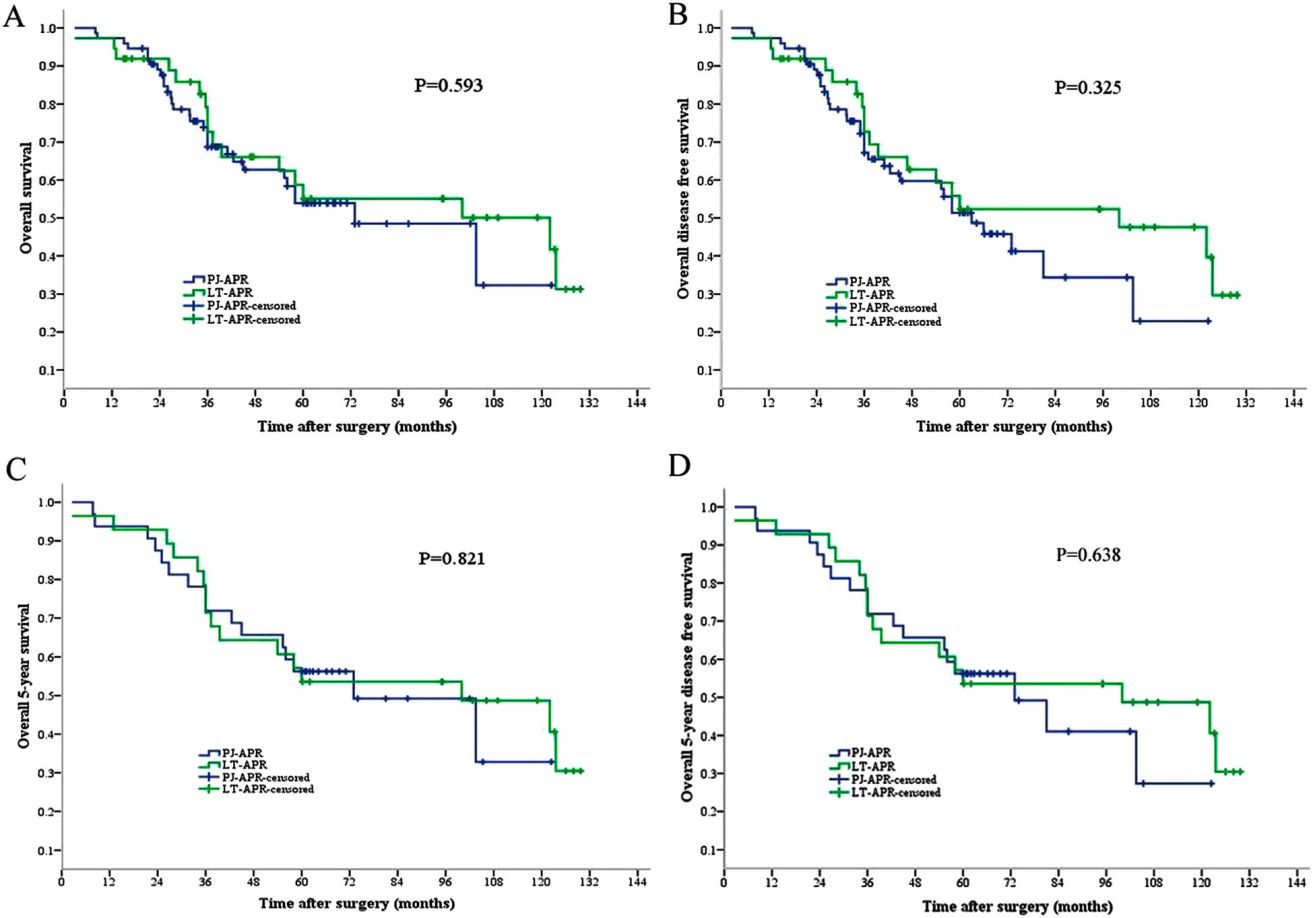
**Kaplan-Meier estimates. (A)** Overall survival of LT-APR vs. PJ-APR (*P* = 0.593). **(B)** Overall disease-free survival of LT-APR vs. PJ-APR (*P* = 0.325). **(C)** Overall 5-year survival of LT-APR vs. PJ-APR (*P* = 0.821). **(D)** Overall 5-year disease-free survival of LT-APR vs. PJ-APR (*P* = 0.638). *PJ-APR* abdominoperineal resection in prone jackknife position, *LT-APR* abdominoperineal resection in lithotomy position.

There were 44 (59.5%) patients alive in the PJ-APR group after a median follow-up of 38.5 (7.8 to 122.4) months, and 20 (54.1%) patients in the LT-APR group were alive after a median follow-up of 47.4 (2.6 to 129.7) months (*P* = 0.593) (Table 3 and Figure 1A).

**Table 3:** **Oncological outcomes according to patient positioning**

**Oncological outcomes**	**PJ-APR**	**LT-APR**	***P*** **value**
Survival	59.5% (44/74)	54.1% (20/37)	0.593
Disease-free survival	52.7% (39/74)	51.4% (19/37)	0.325
Local recurrence	17.6% (13/74)	18.9% (7/37)	0.995
Distant metastasis	23.0% (17/74)	24.3% (9/37)	0.811
5-year survival	50.0% (16/32)	42.9% (12/28)	0.821
5-year disease-free survival	46.9% (15/32)	42.9% (12/28)	0.638
5-year local recurrence	9.4% (3/32)	21.4% (6/28)	0.173
5-year distant metastasis	21.9% (7/32)	28.6% (8/28)	0.382

*PJ-APR* abdominoperineal resection in prone jackknife position, *LT-APR* abdominoperineal resection in lithotomy position.

The overall disease-free survival was 52.7% (39/74) in the PJ-APR group and 51.4% (19/37) in the LT-APR group (*P* = 0.325) (Table 3 and Figure 1B). The rates of local recurrence and distant metastasis were 17.6% (13/74) and 23% (17/74) in the PJ-APR group, and 18.9% (7/37) and 24.3% (9/37) in the LT-APR group (*P* > 0.05, Table 3).

Between January 2002 and February 2008, 60 patients who underwent APR had a follow-up of more than 5 years. Among this subgroup, the overall 5-year survival was 46.7% (28/60), and the overall 5-year disease-free survival was 45% (27/60). There was no significant difference in the overall 5-year survival between the PJ-APR (50%, 16/32) and LT-APR groups (42.9%, 12/28; *P* = 0.821) (Table 3, Figure 1C), as well as for the 5-year disease-free survival rate (46.9% vs. 42.9%; *P* = 0.638) (Table 3, Figure 1D).

The rates of local recurrence (9.4%) and distant metastasis (21.9%) in the PJ-APR group were not significantly different from those in the LT-APR group (21.4% and 28.6%; all *P* > 0.05) (Table 3).

**Figure 2:**
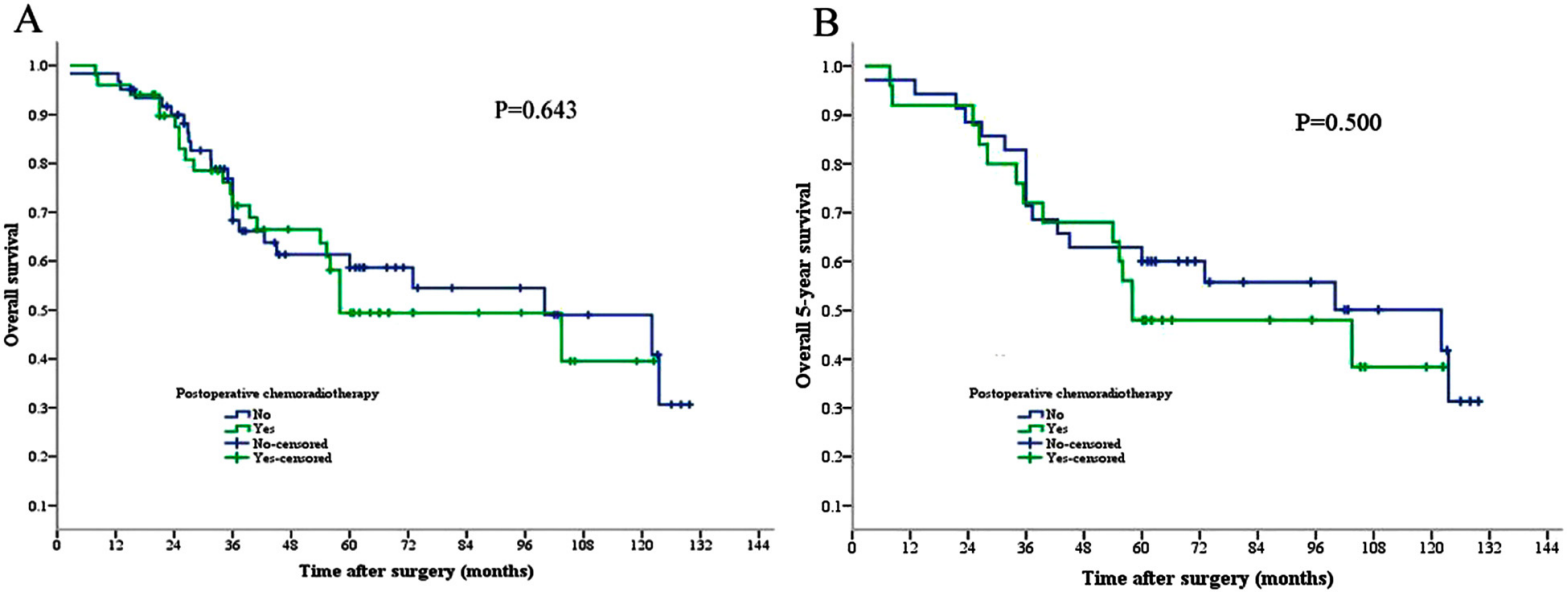
**Chemoradiotherapy and oncological outcomes. (A)** Postoperative chemoradiotherapy and oncological outcomes (*P* = 0.643). **(B)** Postoperative chemoradiotherapy and 5-year oncological outcomes (*P* = 0.500). *PJ-APR* abdominoperineal resection in prone jackknife position, *LT-APR* abdominoperineal resection in lithotomy position.

Fifty-two patients received postoperative radiation therapy and/or chemotherapy. The Kaplan-Meier analysis indicated that postoperative therapy did not affect the overall survival rate in all patients (*P* = 0.643, Figure 2A), or in the subgroup with a follow-up of at least 5 years (*P* = 0.500, Figure 2B).

Cox regression analysis identified gender and pathologic N stage as independent prognostic factors, but operative position of the patient was not a significant factor in this analysis (Table 4).

**Table 4:** **Cox regression analysis of factors associated with survival**

**Clinicopathologic factors**		**HR**	**95% CI**	***P*** **value**
Gender	Male	1		
	Female	0.532	0.385–0.976	0.035
Pathologic N stage	N0	1		
	N1	4.147	2.377–5.890	0.021
	N2	6.738	2.592–18.661	0.002

## Discussion

APR is a complex and challenging surgical procedure. The present study compared the clinical, pathological, and oncological outcomes of PJ-APR vs. LT-APR in low rectal cancer patients in order to confirm which position will provide more benefits to patients undergoing APR. The present study showed that the PJ position was superior to the LT position in terms of operative outcomes. Indeed, compared with LT-APR, the duration of anesthesia and surgery was shorter by >50 min, estimated blood loss in PJ-APR was reduced by >100 mL, and fewer patients needed blood transfusions with PJ-APR. Furthermore, postoperative hospitalization was significantly shorter with PJ-APR, and some of the postoperative complications were significantly reduced with PJ-APR. There was no difference in oncological outcomes between PJ-APR and LT-APR including overall survival, disease-free survival, local recurrence, and distant metastases. These results are supported by previous studies [14].

Perineal dissection is complicated in APR, and performing APR in the PJ position improved visualization, reduced the risks related to the operation, and made the operation easier to perform during this difficult part. Although the LT position provides adequate access to the rectovaginal septum and allows easy access to the posterior face of the rectum [10], this position is uncomfortable for the surgeons and assistants, blood tends to accumulate in the operation area, and adequate lighting is often challenging. Therefore, the surgeon has to spend more time for exposure, operative vision, and hemostasis and also to avoid iatrogenic damage to nerves, blood vessels, and organs. Especially, the anterior wall of the rectum is more difficult to access. Although the patient position needs to be changed during the operation in PJ-APR, this does not need a lot of time [14]. The PJ position is appropriate for almost all proctological surgeries, it allows an excellent exposure of the posterior and anal perineum and the anterior face of the rectum, provides a more comfortable position for the surgeon and assistants, results in less blood accumulation, and enables better lighting [10]. Our results showed that the time needed to change the operation position during PJ-APR was less than 18 min (data not shown); more time was saved in the process of perineal dissection. Moreover, data also showed that the estimated blood loss in PJ-APR was reduced by more than 120 mL compared with LT-APR and that the rate of patients needing blood transfusion was 6% during PJ-APR compared with 14% during LT-APR. In addition, postoperative hospitalization was significantly shorter after PJ-APR compared with LT-APR, which may indicate that patients recovered faster after PJ-APR than LT-APR.

APR requires a complex positioning and exposes the patients to the risks of a major surgery, as well as to the risks specific to this procedure [19]. Previous studies have shown that the incidence of intraoperative perforations was markedly reduced with PJ-APR compared with supine APR [12] and that intraoperative bowel perforation in extralevator APR was significantly reduced when the perineal dissection was performed in the PJ position rather than in the LT or Lloyd-Davies position [11]. The most common immediate complication is intra-abdominal or pelvic abscesses, which has been reported to account for 32% of postoperative complications [20]. The incidence of pelvic abscesses has been estimated at 3% among patients who underwent APR, and it is generally recognized that a pelvic abscess requires reoperation or readmission more frequently than perineal wound infection [21]. Other studies have shown that the risk of postoperative sexual or urinary dysfunction ranges from 10% to 60% [14,22].

In the present study, the incidence of perineal wound infection and incision dehiscence was 1.4% in the PJ-APR group compared with 8%–16% in the LT-APR group. The incidence of pelvic abscesses was also lower with PJ-APR (1.4%) than with LT-APR (2.7%). These results are supported by a previous study (11% vs. 36%) [13], but a much lower incidence was observed in the present study. This low postoperative complication rate may be explained, at least in part, by the fact that all patients underwent curative APR, and not palliative surgery. The other reason is that patients with comorbidities were excluded. In addition, our patients did not receive any neoadjuvant therapy, which could partially explain the low rate of infection. Previous studies have reported infection rates as high as 50% and substantial morbidity, especially after neoadjuvant radiation and chemoradiation therapy [23,24].

Reported survival and local recurrence rates after APR for rectal cancer vary widely. APR is generally associated with poor oncological outcomes [4,25,26]. Indeed, the 5-year cancer-specific survival after sphincter-sparing resection was 74% compared with 62% after APR [27]. However, the same group showed that 2 years after surgery, local recurrence rates were similar with both procedures [28].

A large database analysis suggested that there has been no improvement in mortality following APR from 1996 to 2004 [1]. In the present study, the perineal phase of APR was carried out in the PJ position, followed by abdominal surgery. Because the inferior mesenteric artery is not ligated during the same procedure phase, we were concerned that this may potentially facilitate arterial transport of cancer cells, resulting in metastasis and poor prognosis. However, results showed that overall survival, 5-year overall survival, and oncological outcomes were comparable between the two groups. Cox regression analysis excluded the operative position as being an independent prognostic factor, while gender and pathologic N stage were independent factors associated with survival. Notably, there were no significant differences in pathologic T or N stages, or in the degree of differentiation. However, the number of patients with pT3 and/or poorly differentiated tumors was slightly higher in the PJ-APR group.

The present study suffers from some limitations. First, we were unable to account for the CRM, which has previously been associated with increased local recurrence and reduced patient survival [29-31]. Second, we included all histological subtypes of rectal cancer. However, adenocarcinoma largely predominates (98.3%), and we do not think that the few cases of other subtypes could impact the results much. Third, because of the retrospective nature of the study, we are unable to obtain reliable data about the extralevator approach. Finally, each surgeon selected the operative position for each specific patient based on its appreciation of a number of factors, but there were no specific criteria for LT or PJ. In addition, for a specific patient, a surgeon may have selected the LT position, while another surgeon would have selected the PJ position. Therefore, we cannot provide any exact criteria. We agree that it could have introduced a bias, but results showed that the patients were comparable for most characteristics at baseline.

## Conclusions

The present study suggests that the PJ position provides a better exposure for low rectal cancer and that the operative risks and complications were reduced by performing APR in the PJ position compared with the more conventional LT position. However, the oncological outcomes were not significantly improved. Therefore, PJ-APR might be a better choice for patients with low rectal cancer to reduce operative risks and complications.
